# The Flux of Phenolic Compounds through Silicone Membranes

**DOI:** 10.3390/pharmaceutics5030434

**Published:** 2013-08-21

**Authors:** John Prybylski, Kenneth B. Sloan

**Affiliations:** Department of Medicinal Chemistry, University of Florida, P.O. Box 100485, Gainesville, Florida 32610, USA; E-Mail: jprybylski.rx@ufl.edu

**Keywords:** maximum flux, silicone membrane, Roberts–Sloan equation, water solubility, lipid solubility, human skin *in vitro*

## Abstract

Phenols as a class of molecules have been reported to exhibit higher log maximum fluxes through human stratum corneum, SC, from water, log *J*_MHAQ_, than other classes of molecules. This suggests that their corresponding log maximum fluxes through silicone from water, log *J*_MPAQ_, may be useful to extend the existing *n* = 63 log *J*_MPAQ_ database to include more log *J*_MPAQ_ values greater than 0.0. The log *J*_MPAQ_ values for *n* = 7 phenols predicted to give log *J*_MPAQ_ values greater than 0.0 based on their log *J*_MHAQ_ values have been experimentally determined. These *n* = 7 new log *J*_MPAQ_ values have been added to the existing *n* = 63 log *J*_MPAQ_ database to give a new *n* = 70 database and the *n* = 7 literature log *J*_MHAQ_ values have been added to the existing *n* = 48 log *J*_MHAQ_ database (matched to the *n* = 63 log *J*_MPAQ_ database) to give a new *n* = 55 database. The addition of the *n* = 7 phenols improved the correlations of these flux databases when fitted to the Roberts-Sloan equation, RS, as well as the correlation between the matched experimental (Exp.) log *J*_MPAQ_ with the Exp. log *J*_MHAQ_.

## 1. Introduction

The rate-limiting barrier to diffusion of molecules through human skin is the stratum corneum, SC. The SC is comprised of highly dense, polar, proteinous corneocytes embedded in a lipid matrix. The lipid matrix in turn is comprised of multiple lipid bilayers containing mostly ceramides, fatty acids and cholesterol [1,2,3]. A tortuous pathway through this lipid matrix and around the corneocytes is generally considered to be the route followed by molecules diffusing through the SC. Since the route of molecules diffusing through the SC is lipid-like, the lipid solubility of molecules diffusing through the SC is an important physiochemical determinant of the efficiency of the diffusion process. Similarly, any surrogate for the SC must present with substantially lipid-like properties. Since silicone membranes present with highly lipid-like properties, it has been suggested that silicone membranes could be a surrogate for skin in diffusion cell studies and that those results could be used to predict diffusion through human skin [4,5]. Also, from a theoretical basis it can be assumed that, if the diffusion of unit mass per unit area per unit time, flux, through silicone membranes can be accurately modeled by an equation (Roberts–Sloan, RS, [6]) that accurately models maximum flux through human skin [7] exists, a linear relationship exists between the maximum flux of molecules through silicone membranes from water, log *J*_MPAQ_, and their maximum flux through human skin *in vitro* from water, log *J*_MHAQ_ [5]. Thus experimental (Exp.) log *J*_MPAQ_ could be used to predict Exp. log *J*_MHAQ_. 

The flux data for molecules from which the Roberts–Sloan, RS, equation was derived is based on the application of saturated solutions (suspensions of molecules in a solvent) to the membrane being used to give maximum flux, *J*_M_. Thus all the molecules are presented to the membrane at their maximum thermodynamic activity in that solvent [6,7,8]. Since the molecules are presented to the membrane at their maximum thermodynamic activity, at equilibrium the molecules are also at their maximum thermodynamic activity in the membrane [8,9], *i.e.*, at their solubility limit or saturated solubility in the first few layers of the membrane, *S*_M1_. Thus, *J*_M_ depends only on the solubility of the molecules in the first layers of the membrane, and unless the solvent changes the solubility limit of the membrane, the solvent/vehicle has no effect on *J*_M_. The form of the RS equation for predicting *J*_M_ derives from an expansion of Fick’s law, Equation 1, so that the dependent variables are molecular weight, MW, solubility in a lipid, *S*_LIPID_, and solubility in water, *S*_AQ_. *S*_M1_ can then be estimated from the product of the partition coefficient between the vehicle (water in this case, AQ) and a surrogate lipid for the membrane (octanol in this case, *S*_OCT_), (*K*_OCT:AQ_)*^y^*∙constant, and the solubility in the vehicle, *S*_AQ_: (*K*_OCT:AQ_)*^y^*∙constant∙*S*_AQ_. Expansion of that product into solubilities and taking the logs gives: log *S*_M1_ = *y* log *S*_OCT_ − *y* log *S*_AQ_ + log *S*_AQ_ + log constant = *y* log *S*_OCT_ + (1 − *y*) log *S*_AQ_ + log constant.

*J* = *D*/*L* (*C*_M1_ - *C*_Mn_ ): *J*_M_ = *D*/*L* (*S*_M1_ - *C*_Mn_) (1)

where *D* is the diffusion coefficient of the molecule in the membrane, *L* is the thickness of the membrane, *C*_M1_ is the concentration of the molecule in the first few layers of the membrane and *C*_Mn_ is the concentration in the last few layers of the membrane which is assumed to approach zero. A linear relationship must exist between log *D* + log *S*_M1_ of molecules in a silicone membrane and log *D* + log *S*_M1_ of molecules in human skin in order for a linear relationship between log *J*_MPAQ_ and log *J*_MHAQ_ to exist. 

One problem with determining if the linear relationship between log *J*_MPAQ_ and log *J*_MHAQ_ exists is that there are only about *n* = 63 molecules for which log *J*_MPAQ_ (output) and the necessary corresponding physicochemical properties (log *S*_AQ_ and log *S*_OCT_, input) literature values exist which can be fitted to RS [5]. Of those *n* = 63, only 18 molecules exhibit high output values; log *J*_MPAQ_ values greater than 0.0. Simple phenols present an opportunity to extend the existing *n* = 63 log *J*_MPAQ_ database to include more log *J*_MPAQ_ values greater than 0.0. The log *J*_MHAQ_ values for *n* = 18 phenols and their corresponding physicochemical properties that are necessary to determine their fit to RS were published by Roberts, *et al.* in 1977 [10]. The fit of the *n* = 18 phenols to RS in the fit of the *n* = 62 edited Flynn database to RS was published in 2007 [7]. In the edited Flynn database only *n* = 16 of the *n* = 62 molecules exhibited log *J*_MHAQ_ values greater than 0.0 and of those *n* = 16, *n* = 11 were from among the *n* = 18 phenol subset [10]. Thus, phenols as a subset represent molecules that exhibit physicochemical properties (input) that give higher flux (output) than other types of molecules give.

At present, only *n* = 6 of the *n* = 18 simple phenol subset from Roberts, *et al.* [10] have been included in the *n* = 63 log *J*_MPAQ_ database and only *n* = 2 exhibit log *J*_MPAQ_ greater than 0.0. In order to improve the correlation of the *n* = 63 log *J*_MPAQ_ database with a matched *n* = 48 log *J*_MHAQ_ database, the number of log *J*_MPAQ_ and log *J*_MHAQ_ greater than 0.0 in each database should be increased. Hence, *n* = 7 additional phenols have been selected from the *n* = 18 subset which exhibit physicochemical properties (input) for which RS predicts high log *J*_MPAQ_ values (output). In addition, the *n* = 7 phenols exhibit an average log *J*_MHAQ_ value significantly greater than that of the *n* = 48 log *J*_MHAQ_ database: means ± 95% confidence intervals of 0.04 ± 0.42 log units and −1.06 ± 0.31 log units, respectively. Given the increased range and total number of entries resulting from the addition of these *n* = 7 compounds to the *n* = 48 log *J*_MHAQ_ database and the *n* = 63 log *J*_MPAQ_ database, the fit of these databases to the RS should improve, and correlation of the log *J*_MPAQ_ with log *J*_MHAQ_ values matched in these databases should also improve.

Further, since the addition of the *n* = 7 new entries to the new *n* = 63 log *J*_MPAQ_ database, each potentially exhibiting higher log *J*_MPAQ_ values than the average of the initial *n* = 63 log *J*_MPAQ_ values, will change the relative distribution of flux values in the database, it is imperative to determine if other models would then fit the database better than they did before the addition of the *n* = 7 new entries. Thus, we will also determine the fit of the new databases to the Kasting–Smith–Cooper (KSC) model [11] and to the Magnusson–Anissimov–Cross–Roberts (MACR) model [12] and compare these fits to the fit of RS to the new databases.

## 2. Materials and Methods

The phenolic compounds used are listed in Table 1. These compounds were obtained from Aldrich and their solubility values were acquired or approximated from literature sources. The phenols were all solids except for 3-methylphenol.

The measurement of maximum flux through silicone was performed according to a literature procedure [13] at 32 °C, except that the silicone membrane was in contact with the receptor for only 24 h to condition them. The receptor was a 7.1 pH phosphate buffer.

The donor suspensions were prepared by stirring approximately 0.5 g (1 g in the case of 3-methylphenol) of the compounds in 10 mL of water for 24 h. For all compounds, this surpassed the aqueous solubility by a factor of at least 20, which ensured saturation and excess solid/oil present in the donor phase. After the membranes were conditioned, the receptor phases were changed and the donor suspensions (first application, 1 mL) were applied; *n* = 3. The donor cells were sealed by Parafilm. Samples were taken from the receptor every 2–3 h after application. Following sample collection, the receptor phases were changed to ensure sink conditions, and the donor suspensions were either changed or had more solid/oil added to the existing suspension, depending upon the visible extent of depletion. After 4–5 sampling intervals, the donor suspensions were removed with methanol and the receptors were changed. The membranes were leached with methanol in the donor phase for 48–72 h with samples taken and receptor phases changed every 12–24 h to remove any residual phenol in the membrane.

To ensure that flux data was not altered by possible membrane damage, a standard solute/solvent was applied and its flux determined. A donor suspension was prepared from 400 mg of theophylline suspended with stirring in 6 mL of propylene glycol (PG) for 24 h. This suspension (second application, 0.50 mL) was applied to all the silicone membranes after they were leached with methanol (see above). Samples were taken from the receptor every 24 h after application for at least 72 h so that at least 3 samples were obtained. Following sample collection, the receptor phases were changed and the donor suspensions were changed every other sampling interval. After 3–5 sampling intervals, the diffusion cells were disassembled and the membranes were placed in a methanol bath for maintenance leaching.

The flux values of the first and second application were determined by UV absorption. The wavelengths (λ_ε_) and molar absorptivities (ε) used for the phenolic compounds are listed in Table 1. The log flux of theophylline through silicone from PG, log *J*_MPPG_, for each membrane was found to be within the standard deviation of the literature value of −2.68 ± 0.12 log units [13].

Nonlinear regression was performed by SPSS 20.0 (Rel. 20.0.0). The compounds were fitted to the Roberts–Sloan equation for maximum flux, log *J*_MAQ_:

log *J*_MAQ_ = *x* + *y* log *S*_OCT_ + (1 − *y*) log *S*_AQ_ − *z* MW (2)

to the KSC Equation:

log *J*_MAQ_ = *x* + *y* log *S*_OCT_ − *z* MW (3)

and to the MACR Equation:

log *J*_MAQ_ = *x* − *z* MW (4)

## 3. Results and Discussion

The results are displayed in Table 1. All but 4-chloro-3,5-dimethylphenol exhibited a log *J*_MPAQ_ greater than 0.0, and even it was very close. As a subset the *n* = 7 simple phenols had an average log *J*_MPAQ_ significantly greater than the average log *J*_MPAQ_ of the *n* = 63 log *J*_MPAQ_ database: means ± 95% confidence intervals 1.03 ± 0.45 log units and −0.42 ± 0.29 log units, respectively. The average log *J*_MPAQ_ in the *n* = 70 log *J*_MPAQ_ database has not significantly increased, but is no longer significantly less than 0.0: mean ± 95% confidence interval −0.27 ± 0.29. Unfortunately, the addition of the *n* = 7 phenols did not significantly increase the average log *J*_MHAQ_ of the *n* = 55 log *J*_MHAQ_ database relative to the *n* = 48 log *J*_MHAQ_ database: means ± 95% confidence intervals, −0.918 ± 0.29 log units and −1.058 ± 0.31 log units, respectively.

**Table 1:** The relevant measured or literature physicochemical properties for the *n* = 7 phenolic compounds used in this study.

Cmpd. ^a^	MW	Log *S*_AQ_^b,d^	Log *K*_OCT:AQ_^b^	Log *S*_OCT_^b,d^	λ_ε_^c^	ε ^c,e^	Log *J*_MPAQ_^c,f^	Log *J*_MHAQ_^b,f^
1	143	1.55	3.10	4.65	283	1241	1.01	0.29
2	157	0.28	3.39	3.67	285	1041	−0.027	−0.95
3	122	1.61	2.35	3.96	277	1668	1.37	0.17
4	108	2.29	1.95	4.24	276	1614	1.62	0.53
5	163	1.49	3.08	4.57	285	1791	1.16	0.27
6	197	0.66	3.69	4.35	312	4518	0.49	−0.57
7	108	2.36	1.96	4.32	271	1468	1.61	0.54

^a^ Substituted phenols. 1, 4-chloro-3-methyl; 2, 4-chloro-3,5-dimethyl; 3, 3,4-dimethyl; 4, 4-methyl; 5, 2,4-dichloro; 6, 2,4,6-trichloro; 7, 3-methyl; ^b^ From Roberts *et al.* 1977 [10] and Majumdar *et al.* 2007 [7]; ^c^ Measured directly. ^d^ Solubility in water (*S*_AQ_) or octanol (*S*_OCT_) in μmole cm^−3^; ^e^ Molar absorptivity coefficient in L mole^−1^ cm^−1^; ^f^ Maximum flux through silicone (*J*_MPAQ_) or human stratum corneum (*J*_MHAQ_) from water in μmole cm^−2^ h^−1^.

The addition of these *n* = 7 phenols to the *n* = 63 log *J*_MPAQ_ database and the *n* = 48 log *J*_MHAQ_ database improved the fit of these databases to the RS as expected. The fit of the new *n* = 70 log *J*_MPAQ_ database gave an *r*^2^ of 0.907, an average absolute residual log *J*_MPAQ_ (Δlog *J*_MPAQ_) of 0.300 log units and the coefficients *x* = −1.606, *y* = 0.695 and *z* = 0.00490 were all significant (*p* < 0.05):

log *J*_MPAQ_ = −1.606 + 0.695 log *S*_OCT_ + 0.305 log *S*_AQ_ − 0.00490 MW (5)

The fit of the *n* = 70 log *J*_MPAQ_ database is an improvement over the *n* = 63 log *J*_MPAQ_ database, which had *r*^2^ = 0.896 and Δlog *J*_MPAQ_ = 0.310 log units, but had similar coefficient values: *x* = −1.607, *y* = 0.701, *z* = 0.00492. The fit of the new *n* = 55 log *J*_MHAQ_ database gave an *r*^2^ of 0.883, an average absolute residual log *J*_MHAQ_ (Δlog *J*_MHAQ_) of 0.282 log units and the coefficients *x* = −3.005 and *y* = 0.654 were significant (*p* < 0.05), but the coefficient *z* = 0.00112 was not significant (*p* = 0.25):

log *J*_MHAQ_ = −3.005 + 0.645 log *S*_OCT_ + 0.346 log *S*_AQ_ − 0.00112 MW (6)

**Figure 1:**
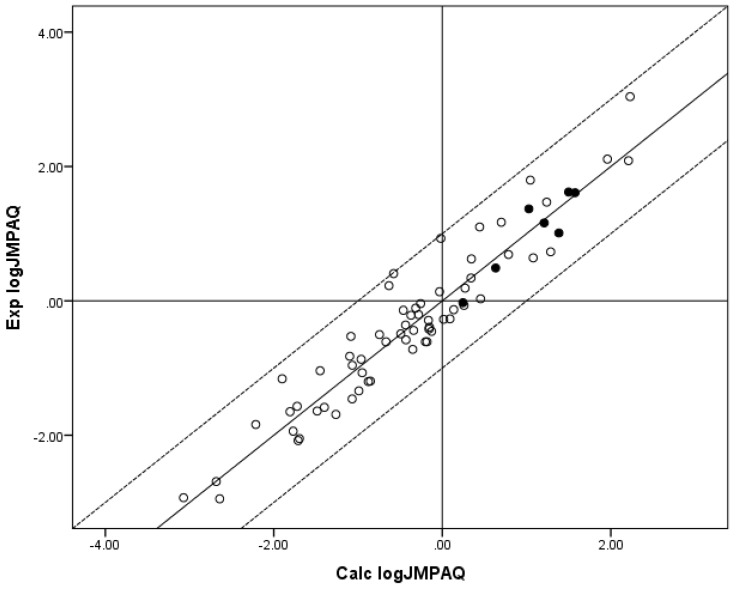
The correlation of the calculated (Calc.) log *J*_MPAQ_ from the fit of *n* = 70 to RS with the experimental (Exp.) log *J*_MPAQ_. The dashed lines represent the boundaries for residual log *J*_MPAQ_ greater than 1.0, the solid line indicates points where the Calc. log *J*_MPAQ_ is equivalent to the Exp. log *J*_MPAQ_. The filled circles indicate the *n* = 7 phenols. The Calc. log *J*_MPAQ_ values were determined with Equation 5: log *J*_MPAQ_ = −1.606 + 0.695 log *S*_OCT_ + 0.305 log *S*_AQ_ − 0.00490MW. *r*^2^ = 0.907, average absolute residual log *J*_MPAQ_ = 0.300.

**Figure 2:**
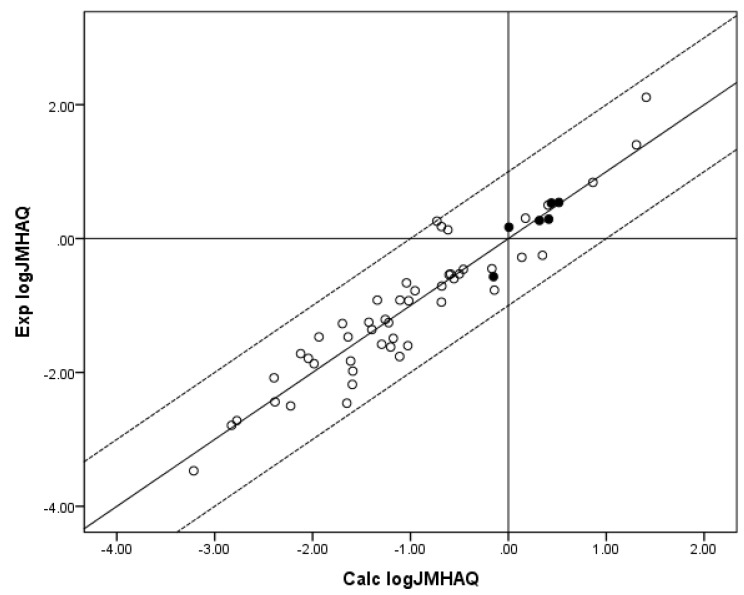
The correlation of the calculated (Calc.) log *J*_MHAQ_ from the fit of *n* = 55 to RS with the experimental (Exp.) log *J*_MHAQ_. The dashed lines represent the boundaries for residual Exp. log *J*_MHAQ_ greater than 1.0, and the solid line indicates points where the Calc. log *J*_MHAQ_ is equivalent to the Exp. log *J*_MHAQ_. The filled circles indicate the *n* = 7 phenols. The Calc. log *J*_MHAQ_ values were determined with Equation 6: log *J*_MHAQ_ = −3.005 + 0.654 log *S*_OCT_ + 0.346 log *S*_AQ_ − 0.00112 MW, *r*^2^ = 0.883, average absolute residual log *J*_MHAQ_ = 0.282.

The lack of statistical significance for the *z* coefficient indicates a need to further extend the *n* = 55 log *J*_MHAQ_ database, since the significance of MW to maximum flux is well-established [12]. The fit of the *n* = 55 log *J*_MHAQ_ database to the RS is an improvement over the *n* = 48 log *J*_MHAQ_ database, which had *r*^2^ = 0.867 and Δlog *J*_MHAQ_ = 0.331 log units and the coefficients *x* = −2.763, *y* = 0.635 and *z* = 0.00207. The *x* and *y* coefficients for the new log *J*_MHAQ_ database are substantially closer to those coefficients determined for the *n* = 62 edited Flynn log *J*_MHAQ_ database: *x* = −3.008, *y* = 0.732, *z* = 0.0048. Relevant results are displayed in Table 2 and Figure 1, Figure 2. Figure 1 shows a plot of experimental (Exp.) log *J*_MPAQ_* versus* log *J*_MPAQ_ calculated (Calc.) from the coefficients for the fit of the *n* = 70 database to RS, and Figure 2 shows a plot of Exp. log *J*_MHAQ_* versus* log *J*_MHAQ_ Calc. from the coefficients for the fit of the *n* = 55 database to RS. 

**Table 2:** The calculated (Calc.), predicted (Pred.), and experimental (Exp.) maximum flux values through silicone from water (log *J*_MPAQ_) and through human stratum corneum from water (log *J*_MHAQ_) for the *n* = 7 phenolic compounds.

Cmpd. ^a^	Exp. log *J*_MPAQ_^b^	Pred. *n* = 63 log *J*_MPAQ_^b,c^	Calc. *n* = 70 log *J*_MPAQ_^b,d^	Exp. log *J*_MHAQ_^b^	Pred. *n* = 48 log *J*_MHAQ_^b,e^	Calc. *n* = 55 log *J*_MHAQ_^b,f^
1	1.01	1.41	1.40	0.29	0.46	0.41
2	−0.027	0.28	0.26	−0.95	−0.66	−0.68
3	1.37	1.05	1.04	0.17	0.087	0.0053
4	1.62	1.52	1.51	0.53	0.54	0.44
5	1.16	1.24	1.23	0.27	0.35	0.32
6	0.49	0.67	0.65	−0.57	−0.17	−0.15
7	1.61	1.60	1.59	0.54	0.62	0.52
Δlog *J*_MAQ_^g^		0.200	0.195		0.159	0.162

^a^ Substituted phenols. 1, 4-chloro-3-methyl; 2, 4-chloro-3,5-dimethyl; 3, 3,4-dimethyl; 4, 4-methyl; 5, 2,4-dichloro; 6, 2,4,6-trichloro; 7, 3-methyl; ^b^ Given in units μmole cm^−2^ h^−1^; ^c^ Using RS coefficients *x* = −1.607, *y* = 0.701, *z* = 0.00492 [6]; ^d^ Using RS coefficients *x* = −1.606, *y* = 0.695, *z* = 0.00490; ^e^ Using RS coefficients *x* = −2.763, *y* = 0.635 and *z* = 0.00207 [6]; ^f^ Using RS coefficients *x* = −3.005, *y* = 0.654, *z* = 0.00112; ^g^ Average absolute residual log *J*_MPAQ_ or log *J*_MHAQ_ for the *n* = 7 phenols.

Plots of the individual independent variables, log *S*_OCT_, log *S*_AQ_ and MW, against log *J*_MPAQ_ (*n* = 70) and against log *J*_MHAQ_ (*n* = 55) gave the following *r*^2^ values: (a) log *J*_MPAQ_* versus* log *S*_OCT_, *r*^2^ = 0.677; *versus* log *S*_AQ_, *r*^2^ = 0.554; *versus* MW, *r*^2^ = 0.541; (b) log *J*_MHAQ_* versus* log *S*_OCT_, *r*^2^ = 0.603; *versus* log *S*_AQ_, *r*^2^ = 0.526; *versus* MW, *r*^2^ = 0.520. All regression equations had statistically significant (*p* < 0.05) slope and intercept estimates. In each case, the best regression of the individual independent variables against flux values was by log *S*_OCT_. It is worth noting that the *n* = 7 phenol subset gives the following *r*^2^ values and significance profiles when the individual independent variables log *S*_OCT_ and log *S*_AQ_ are plotted against log *J*_MPAQ_ and log *J*_MHAQ_: (a) log *J*_MPAQ_* versus* log *S*_OCT_, *r*^2^ = 0.180, without a statistically significant slope (*p* = 0.34) or intercept (*p* = 0.51); *versus* log *S*_AQ_, *r*^2^ = 0.949, with a statistically significant slope (*p* < 0.05), but without a statistically significant intercept (*p* = 0.50); (b) log *J*_MHAQ_* versus* log *S*_OCT_, *r*^2^ = 0.335, without a statistically significant slope (*p* = 0.17) or intercept (*p* = 0.18); *versus* log *S*_AQ_, *r*^2^ = 0.933, with statistically significant (*p* < 0.05) slope and intercept. The improved dependence of maximum flux from water on the aqueous solubility of highly water-soluble compounds is demonstrated here as a property of both silicone and human stratum corneum, and will be a topic of future investigations. When two individual independent variables, log *S*_OCT_ and MW, from the *n* = 70 log *J*_MPAQ_ database were fitted to the KSC equation (Equation 3) the following *x*, *y* and *z* coefficients to the parameters were obtained along with *r*^2^ and Δlog *J*_MPAQ_ values: *x* = −0.923, *y* = 0.794, *z* = 0.0089, *r*^2^ = 0.797 and Δlog *J*_MPAQ_ = 0.431. All but the *y* coefficient (*p* = 0.069) were statistically significant (*p* < 0.05):

log *J*_MPAQ_ = −0.923 + 0.794 log S_OCT_ − 0.0089 MW (7)

**Figure 3:**
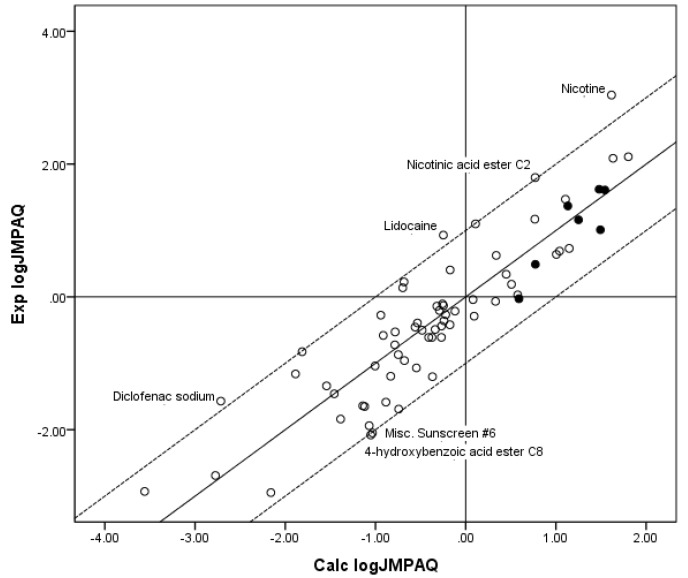
The correlation of the calculated (Calc.) log *J*_MPAQ_ from the fit of *n* = 70 to KSC with the experimental (Exp.) log *J*_MPAQ_. The dashed lines represent the boundaries for residual Exp. log *J*_MPAQ_ greater than 1.0, the solid line indicates points where the Calc. log *J*_MPAQ_ is equivalent to the Exp. log *J*_MPAQ_. The filled circles indicate the *n* = 7 phenols. The Calc. log *J*_MPAQ_ values were determined with Equation 7: log *J*_MPAQ_ = −0.923 + 0.794 log *S*_OCT_ − 0.0089 MW, *r*^2^ = 0.797, average absolute residual log *J*_MPAQ_ = 0.431.

Figure 3 shows a plot of Exp. log *J*_MPAQ_* versus* log *J*_MPAQ_ values calculated from the coefficients for the fit of the *n* = 70 database to Equation 7. Although the *r*^2^ was substantially improved by including MW with log *S*_OCT_ as independent variables in the regression against flux, the *r*^2^ was poorer than the *r*^2^ for the fit of all three independent variables to the Roberts–Sloan Equation (Equation 5). Similarly, when two individual independent variables, log *S*_OCT_ and MW, from the *n* = 55 log *J*_MHAQ_ database were fit to the KSC equation (Equation 8) the following *x*, *y* and *z* coefficients to the parameters were obtained along with *r*^2^ and the Δlog *J*_MHAQ_ values: *x* = −1.252, *y* = 0.602, *z* = 0.0080, *r*^2^ = 0.723 and Δlog *J*_MHAQ_ = 0.441. The estimates for the coefficients were all statistically significant (*p* < 0.05): 

log *J*_MHAQ_ = −1.252 + 0.602 log *S*_OCT_ − 0.0080 MW (8)

**Figure 4:**
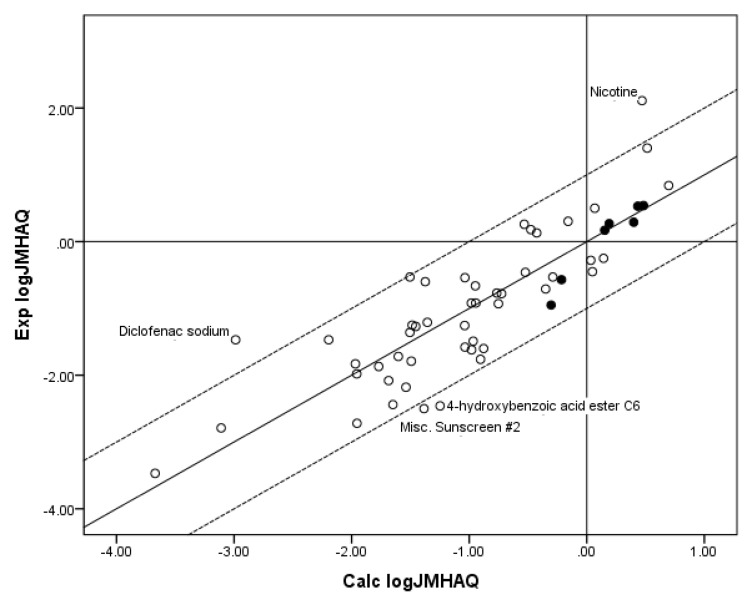
The correlation of the calculated (Calc.) log *J*_MHAQ_ from the fit of *n* = 55 to KSC with the experimental (Exp.) log *J*_MHAQ_. The dashed lines represent the boundaries for residual Exp. log *J*_MHAQ_ greater than 1.0, and the solid line indicates points where the Calc. log *J*_MHAQ_ is equivalent to the Exp. log *J*_MHAQ_. The filled circles indicate the *n* = 7 phenols. The Calc. log *J*_MHAQ_ values were determined with Equation 8: log *J*_MHAQ_ = −1.252 + 0.602 log *S*_OCT_ − 0.0080 MW, *r*^2^ = 0.723, average absolute residual log *J*_MHAQ_ = 0.441.

Figure 4 shows a plot of Exp. log *J*_MHAQ_* versus* log *J*_MHAQ_ values calculated from the coefficients for the fit of the *n* = 55 database to Equation 8. Again, although a substantial improvement in *r*^2^ was obtained by including MW with log *S*_OCT_ as independent variables in the regression against flux, the *r*^2^ was poorer than the *r*^2^ for the fit of all three independent variables to the RS equation (Equation 6). The fit of both databases to the MACR equation (Equation 4), which is the remaining model used to predict maximum flux, is simply the regression of MW against log *J*_MPAQ_ or log *J*_MHAQ_ shown above to give a somewhat poorer fit than regression of the two individual independent variables, log *S*_OCT_ or log *S*_AQ_, against log *J*_MPAQ_ or log *J*_MHAQ_. Finally, it should be noted the popular Potts–Guy Equation [14] was not included as a model because its output is permeability coefficient which is not clinically relevant.

**Figure 5:**
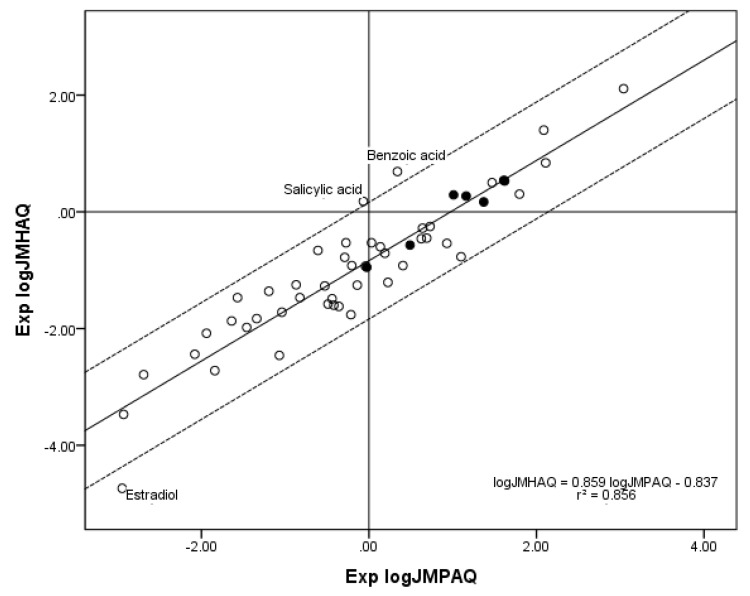
The correlation of the *n* = 52 log *J*_MHAQ_ with log *J*_MPAQ_ database. The dashed lines represent the boundaries for residual Exp. log *J*_MHAQ_ greater than 1.0, and the solid line is the regression equation. The filled circles indicate the *n* = 7 phenols. The regression information is in the figure.

The new *n* = 52 database of compounds contributing to both the *n* = 70 log *J*_MPAQ_ database and the *n* = 55 log *J*_MHAQ_ database also gives a higher correlation between Exp. log *J*_MPAQ_ and Exp. log *J*_MHAQ_ than the previous *n* = 45 database. A linear regression yielded the expression Exp. log *J*_MHAQ_ = 0.859 Exp. log *J*_MPAQ_ − 0.837, *r*^2^ = 0.856, which is an improvement over *r*^2^ = 0.838 for the *n* = 45 database. Figure 5 shows the plot of Exp. log *J*_MPAQ_* versus* Exp. log *J*_MHAQ_ for *n* = 52. 

## 4. Conclusions

The addition of the *n* = 7 phenols improved all aspects of the log *J*_MPAQ_ database with regards to comparison with the matched log *J*_MHAQ_ database. Along with strengthening the validity of the assertion that silicone membranes are good surrogates for human stratum corneum, these improvements demonstrate that this surrogate nature holds for a wider range of log *J*_MPAQ_ and log *J*_MHAQ_ values than had been previously reported. 
